# Understanding surgical antimicrobial prescribing behaviour in the hospital setting: a systematic review and meta-ethnography protocol

**DOI:** 10.1186/s13643-020-01477-5

**Published:** 2020-10-10

**Authors:** Hazel Parker, Julia Frost, Nicky Britten, Sophie Robinson, Karen Mattick

**Affiliations:** 1 Pharmacy Department, Royal Devon and Exeter NHS Foundation Trust, Barrack Road, Exeter, EX2 5DW UK; 2 South Cloisters, University of Exeter, St Luke’s Campus, Heavitree Road, Exeter, EX1 2LU UK

**Keywords:** Antimicrobial decision-making, Antimicrobial prescribing behaviour, Meta-ethnography, Qualitative, Surgery, Synthesis

## Abstract

**Background:**

Surgical specialities use extensive amounts of antimicrobials, and misuse has been widely reported, making them a key target for antimicrobial stewardship initiatives. Interventions informed by, and tailored to, a clear understanding of the contextual barriers to appropriate antimicrobial use are more likely to successfully improve practice. However, this approach has been under utilised. Our aim is to synthesise qualitative studies on surgical antimicrobial prescribing behaviour (APB) in hospital settings to explain how and why contextual factors act and interact to influence APB amongst surgical teams. We will develop new theory to advance understanding and identify knowledge gaps to inform further research.

**Methods:**

The meta-ethnography will follow the seven-phase method described by Noblit and Hare. We will conduct a comprehensive search using eight databases (AMED, CINAHL, EMBASE, MEDLINE, MEDLINE-in-process, Web of Science, Cochrane Library and PsycINFO) with no date restrictions; forwards and backwards citation searches; and contacting first authors of relevant papers. Studies will be dual screened and included if they use recognised qualitative methods and analysis; focus on contextual factors associated with surgical APB within hospital settings; are available in full in English; and are relevant to the research question. Any disagreements between reviewers will be resolved through discussion to reach consensus. Included studies will be read repeatedly to illuminate key concepts and the relationship between key concepts across studies. Then, key concepts will be sorted into conceptual categories or ‘piles’ which will be further abstracted to form a conceptual framework explaining surgical APB. During the synthesis, emerging interpretations will be discussed with stakeholders (including authors of included studies where possible; surgical and stewardship practitioners; and patient representatives) to ensure new knowledge is meaningful.

**Discussion:**

This research has several strengths: (1) the protocol has been written with reference to established guidance maximising rigour and transparency; (2) the multi-disciplinary research team bring varied interpretative repertoires and relevant methodological skills; and (3) stakeholders will be involved to ensure that findings are relevant, and disseminated via suitable channels, to support improved patient care.

**Systematic review registration:**

PROSPERO CRD42020184343

## Background

Antimicrobial resistance (AMR) represents a global patient safety risk. By 2050, AMR will be responsible for an estimated ten million deaths annually [1] unless policies are successfully implemented to tackle its spread. One of the key strategies to reduce AMR is antimicrobial stewardship which encompasses the careful and responsible use of antimicrobials to improve patient outcomes. Antimicrobial misuse—including unnecessary use; wrong dose, route or duration of therapy; wrong agent; delayed administration in critically ill patients and mistimed surgical prophylaxis—leads to increased AMR; hospital-acquired infections; and other antimicrobial-associated adverse events.

Twenty percent of human antimicrobial consumption occurs in hospitals, and over the last 5 years usage has increased by 6.3% (or 2.8% if adjusted for growth in patient admissions over this time) [2]. A high proportion of this prescribing occurs in surgical specialities where inappropriate prescribing has been widely reported [3–7] with numerous calls for action [8–11]. Around ten million patients undergo surgery within the National Health Service (NHS) each year [12] with advances in surgical technique and anaesthesia resulting in more patients, at increased risk of infection, being offered surgery [13]. A single dose of surgical antimicrobial prophylaxis (SAP) is vital for many procedures, usually on starting anaesthesia, to limit surgical site infection [13]. SAP efficacy relies on the availability of suitable agent(s) that ‘cover’ bacteria likely to be encountered during the surgical procedure. Antimicrobial treatment is only indicated if the patient develops an infection, for example, a surgical site infection. However, the prevalence of healthcare-associated infection is high amongst surgical patients (8.5%), second only to intensive care, and 39.5% of surgical patients are prescribed an antimicrobial on any given day [14]. In English hospitals, 1 in 12 patients are administered SAP: about half receive more than the recommended single dose and a third receive more than 24 h of antimicrobial cover [14]. Antimicrobial treatment courses prescribed by surgeons are less likely to be compliant with evidence-based guidelines, compared to treatment courses prescribed in general medicine, and more likely to be escalated to broader spectrum agents (which have activity against a wide range of microorganisms) [15]. This is despite the wide availability of international [16], national [13, 17–20] and local [21] guidelines reinforcing the point that the provision of guidelines alone is insufficient to change practice [22]. There is an urgent need to improve surgical APB to minimise unintended patient harm.

Interventions that are informed by, and tailored to, a clear understanding of the contextual barriers to appropriate antimicrobial use are more likely to improve practice [23, 24]. However, little attention has been paid to how interventions work in different contexts and for different prescribing groups [25] and many interventions do not use effective behaviour change techniques [26]. To bring about meaningful, sustained behaviour change, it is essential to understand the contextual factors influencing surgical APB. Then, holistic, context-sensitive interventions can be co-designed and delivered accordingly, to improve patient care.

Multiple reasons for antimicrobial misuse within surgery have been postulated, including a lack of training, experience or confidence; inadequate knowledge of local AMR epidemiology; misinterpretation of microbiology results; uncertain diagnosis and/or lack of guidance or institutional leadership [8]. Additionally, a growing body of qualitative studies have described surgical teams’ APB, highlighting it as distinct from that of other physicians [27–29]. Surgeons view antimicrobial management as peripheral to their role. Fear of failure, risk of blame and lack of expertise all contribute to inappropriate antimicrobial use [27]. Professional hierarchies within and between specialities influence SAP decisions [30], with obstacles to timely administration including organisational communication, inconvenience, workflow, role perception and the low priority assigned to antimicrobials [31]. Post-operatively, the ward round becomes central to decision-making. However, rounds are often rushed owing to pressure on the surgeons to be in theatre; responsibility for antimicrobial prescribing is often delegated to junior staff; team members are rarely present for the entire round (reducing continuity); and juniors, usually responsible for keeping track of key decisions and tasks, are frequently sent away from the round to chase results, or are omitted from critical conversations. As a result, team members become unclear about which patients are prescribed antimicrobials [27].

A synthesis of qualitative studies on surgical APB is warranted to generate a comprehensive and transferrable theory that will inform future research and antimicrobial stewardship programmes [32]. Meta-ethnography is a well-established qualitative evidence synthesis methodology with origins in the interpretive paradigm. It was first developed by Noblit and Hare (1988) in the field of education, because their aggregative synthesis could not explain the failure of desegregation in schools, but it is now widely used in health and social care research [33–35]. The seven-phase process integrates and compares findings from multiple qualitative studies facilitating the identification of overarching constructs and development of new theory [36]. Overarching practical knowledge in the form of a theory (system of ideas explaining phenomena) has the potential to support healthcare workers and policymakers by providing a complex and comprehensive conceptual understanding of things that cannot be ‘pinned down’ [37]. Our aim is to synthesise qualitative studies on surgical APB in hospital settings to explain how and why contextual factors act and interact to influence APB amongst surgical teams. We will develop new, clinically applicable, theory to advance understanding and identify knowledge gaps to inform further research.

## Methods

**Fig. 1:**
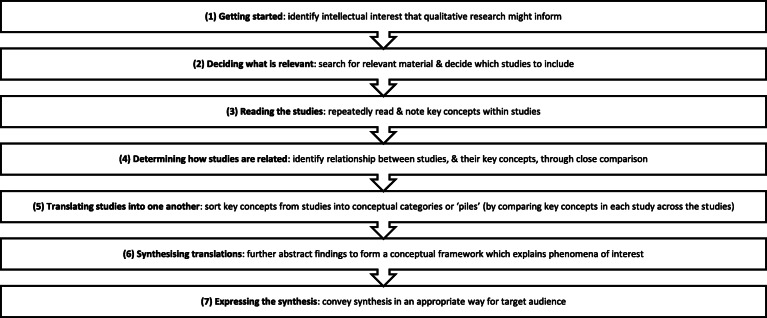
Noblit and Hare’s [36] seven phases of meta-ethnography

The meta-ethnography has been registered with the International Prospective Register of Systematic Reviews (PROSPERO); registration number CRD42020184343. Protocol development was informed by recent advances in meta-ethnographic theory and practice [33, 34, 36, 38–42]; and with reference to the Preferred Reporting Items for Systematic Review and Meta-analysis Protocols (PRISMA-P) checklist (see Additional file). Data from eligible papers will be synthesised following the seven phases (see Fig. 1) outlined by Noblit and Hare; however, meta-ethnography is not a linear process and these phases will likely overlap and repeat as the synthesis proceeds [36]. Findings will be evaluated using the Confidence in Evidence from Reviews of Qualitative Research (CERQual) approach [43], and reported in accordance with the eMERGe reporting guidelines [44]. Ethical approval is not required for a synthesis of published peer-reviewed studies (http://www.hra-decisiontools.org.uk/ethics/).

### Theoretical perspective

We (HP, KM and NB) collaborated to prepare a National Institute for Health Research funding application with the intention to build on previous work [45, 46] and this has now been funded. HP has worked as a National Health Service Hospital Pharmacist for the past 15 years, specialising in the field of antimicrobials since 2009, and is now a National Institute for Health Research (NIHR) Clinical Doctoral Research Fellow. JF and NB are Medical Sociologists with extensive qualitative research and meta-ethnography experience. KM is a Professor of Medical Education and has broad research experience including qualitative methods and analysis. SR is an experienced information specialist.

Meta-ethnography is an inductive, highly interpretive approach [36, 40]. We will be endeavouring to develop new interpretations from first-order data (primary study participant interpretations), and second-order data (author interpretations of participant interpretations) presented in the primary studies. To broaden perspective and to support the development of insightful, practical theory, we have formed a stakeholder group that will contribute to the synthesis. Stakeholders will include a consultant surgeon, anaesthetist and microbiologist; a hospital pharmacist; and several patient representatives. Additionally, technical support will be provided by an information specialist (SR) and a research fellow (EC) from the NIHR Applied Research Collaboration South West Peninsula Patient and Public Involvement team.

### Phase one: getting started

#### Formulating the research question

Our research question is how and why do contextual factors act and interact to influence surgical APB in hospital settings? Several qualitative studies have explored the subject. However, a synthesis offers us a tool to understand this body of work more fully, more deeply and more convincingly [47], whereas other approaches might remove context and/or impede explanation [36]. Meta-ethnography is the preferred qualitative evidence synthesis method as it is systematic and has the potential to preserve interpretive properties from the primary studies [48]. Furthermore, it aims to develop conceptual understanding [36], which aligns with our intentions, and has been implemented previously to develop theory about antimicrobial prescribing interventions in general practice [35]. As far as we know, this will be the first meta-ethnography addressing surgical APB.

### Phase two: deciding what is relevant

#### Focus of the synthesis

We decided to focus on studies that employed recognised qualitative methods and analysis (i.e. primary qualitative or mixed methods studies) to explore APB amongst surgical team members (from any surgical speciality). This is because conceptually rich studies are a pre-requisite for interpretive synthesis methods. Other types of studies (e.g. quantitative questionnaire studies with some open-ended free text questions) would be unlikely to provide the rich elaborations needed to make interpretations and to build understanding. Qualitative studies of APB in primary care, or those that did not differentiate surgical teams from non-surgical specialities in hospital settings, were not included because we view surgeons as a distinct group requiring a nuanced antimicrobial stewardship approach.

#### Locating relevant studies

HP and an information specialist (SR) will develop a search strategy (see Appendix), and will systematically search eight databases (AMED, CINAHL, EMBASE, MEDLINE, MEDLINE-in-process, Web of Science, Cochrane Library and PsycINFO) from their inception to identify potentially relevant studies. This combination of databases will enable representation from a range of domains including medical and allied health professional research and clinical practice; sociology; psychology and related disciplines. This is important as qualitative research is frequently catalogued outside the medical domain. The SPIDER tool [49] (see Table 1) has been used to provide structure for the search, although search terms will be individualised for each database. Because qualitative literature can be challenging to find [50], we will employ supplementary search methods [51] to identify additional suitable studies: (1) forwards and backwards citation searching using studies that meet the inclusion criteria for the meta-ethnography; and (2) we will contact experts in the field, including the authors of all included studies, to ask them to suggest any additional studies (including those in-press).

**Table 1:** SPIDER table of study inclusion and exclusion criteria

	Inclusion criteria	Exclusion criteria
Sample	• Surgical teams (any members including surgeons, trainee surgeons, anaesthetists, surgical nurses, surgical pharmacists etc.) • Secondary care setting including wards; out-patient clinics; theatres etc.	• Non-surgical specialities • Other care settings, e.g. primary care; dentists • Veterinary studies
Phenomenon of interest	• Antimicrobial/antibiotic prescribing behaviour (treatment and/or prophylaxis)	• Prescribing behaviour related to other medication classes
Design	• Qualitative or mixed-method studies reporting primary qualitative data collected using qualitative methods (e.g. through direct observation; focus groups or interviews)	• Studies that report quantitative data only including questionnaire studies with open-ended free text questions
Evaluation	• Qualitative analysis of antimicrobial prescribing behaviour (using any qualitative evaluation, e.g. grounded theory; and framework analysis)	• Studies that evaluate using quantitative methods only • Studies that do not explicitly state the method of analysis
Research type	• Peer-reviewed journal articles • Full text available • English language	• Reviews; protocols; theoretical work; editorials; opinion pieces and grey literature • Non-English language

The purpose of the comprehensive search is to identify the relevant body of literature containing information on the contextual factors associated with surgical APB. A comprehensive approach has been chosen as (1) it will ensure that all relevant work is cited, to facilitate the development of theory and to prevent unhelpful research repetition/waste; and (2) it is more likely to resonate with our target audience (surgical and stewardship teams) who are more familiar with quantitative systematic reviews. Scoping searches suggest that the volume of applicable literature will be manageable. However, should the number of studies uncovered become unwieldy, we will use a purposive or theoretical sampling strategy in keeping with the epistemology of meta-ethnography [50].

#### Inclusion and exclusion decisions

All retrieved studies will be imported into Endnote reference management software and de-duplicated. HP and SR will then independently screen the studies based on the title and abstract.

Studies will be excluded if they do not have a qualitative component or do not describe APB in a surgical context (see Table 1). In the event of uncertainty or disagreement studies will be sought in full, in addition to the studies that definitely appear relevant, to be assessed by HP and one other author (KM or JF). Studies will be included if they use recognised qualitative methods (e.g. interviews, focus groups or observation) and analysis (e.g. framework analysis or thematic analysis); focus on contextual factors associated with surgical (any speciality) APB within a hospital setting, and are available in full in the English language. Any disagreement at the final screening stage will be resolved by consensus between three reviewers (HP, JF and KM).

#### Quality assessment

No studies will be excluded based on quality alone [52]. However, all studies will be assessed using the qualitative Critical Appraisal Skills Programme (CASP) tool [53] to support careful and systematic reading [40] with consideration of a range of aspects [41]. Lower quality assessment scores—for example, due to poor reporting or abridged methods sections (often the case in medical journals)—does not always reflect the quality of the research; however, it can draw the reviewer’s attention to shortcomings in the interpretation of study findings that may have an impact on the results of the synthesis [38].

Three reviewers (HP, KM and JF) will then use a pragmatic approach, first described by Dixon-Woods et al. (2007) [54], to classify studies based on their perceived utility to the meta-ethnography (see Table 2). Those studies deemed ‘irrelevant’ or ‘fatally flawed’ will be excluded. Remaining papers—key papers; satisfactory papers; and questionable papers—will be included in the synthesis. Any disagreement regarding categorisation of a study will be resolved by consensus between the three reviewers. Additionally, synthesis messages derived from the included studies will be examined against ‘key’ papers (only) to test their contributions and promote further discussion and insight, consistent with previous work [41]. A Microsoft Excel spreadsheet will be used to collate study demographics; appraisal scores and inclusion/exclusion decisions.

**Table 2:** Study classifications [54]

Category	Study characteristics
Key papers	Conceptually rich with the potential to make an important contribution to the synthesis
Satisfactory papers	Less valuable than key papers but still relevant
Questionable papers	Uncertain contribution
Irrelevant	Not relevant to the review question
Fatally flawed	Study data not presented in a usable format

### Phase three: reading the studies

This stage of the meta-ethnography involves repeated careful reading of the studies to gain familiarity and to identify the main concepts described, i.e. what is each study telling us. Contextual information, such as study setting; participants (e.g. sub-speciality, grade and number included); research design and aim, will be recorded in a Microsoft Excel spreadsheet. Following repeated close reading (HP, KM and JF), the studies will be imported into the NVivo qualitative data analysis software. Key concepts (potentially explanatory ideas) from each study’s results and/or discussion section will then be independently coded by two reviewers (HP and JF or KM). The coded data will include quotations from participants (first-order data) and quotations from the original study’s authors (second-order data). Reviewers (HP, JF and KM) will then discuss and agree on the key concepts, recording them in a Microsoft Excel spreadsheet.

### Phase four: determining how the studies are related

The next phase requires us to identify the relationship between the primary studies, and their key concepts, through a process of close comparison. We will begin to determine whether the synthesis is ‘reciprocal’ (primary studies’ concepts are directly complementary), ‘refutational’ (primary studies’ concepts oppose each other) or ‘lines-of-argument’. In the latter case, primary studies identify different aspects of a larger phenomenon which when taken together offer a new interpretation; a ‘whole’ is discovered from a set of parts [36]. We are aware that meta-ethnographies frequently produce reciprocal or lines-of-argument translations [55]. Lines of argument syntheses often bring together interrelated concepts, but may also represent a lack of attention to conflicting findings. We will actively seek disconfirming or contradictory findings and concepts.

### Phase five: translating the studies into one another

Translation involves sorting the key concepts (from primary studies) into conceptual categories or ‘piles’ [42]. HP, KM and JF will independently compare key concepts across the primary studies, grouping them all into conceptual categories (third-order data) with a definition of what each conceptual category encompasses. The conceptual categories will be developed inductively, through a process of constant comparison (of key concepts), rather than according to any a priori theory, although we recognise each reviewer’s interpretations will be influenced by their backgrounds. The reviewers will then compare interpretations and, with input from stakeholders, collaboratively develop a final list of conceptual categories, which will be tested against the primary studies to ensure a good fit. The multi-disciplinary input will enable us to challenge our own understandings and will support the identification of a range of possible analytic interpretations.

### Phase six: synthesising translations

Synthesising translations is the on-going process whereby findings are further abstracted to form a conceptual framework [42], which explains the phenomena of interest. It cannot be reduced to a set of mechanistic tasks [39] but will involve three reviewers (HP, KM and JF) working collaboratively to ‘make sense’ of the conceptual categories, with the aim of developing a new theory that explains how and why contextual factors act and interact to influence APB amongst surgical teams. If appropriate, a visual way of representing the findings will be developed iteratively, to convey the theory.

Emerging interpretations will be discussed with (1) the authors of the primary studies (where possible) to test the validity of our third-order interpretations; (2) academic and surgical/stewardship audiences to receive feedback, for example at departmental seminars, conferences and methodological discussion fora and (3) our wider stakeholder group to ensure that the knowledge is applicable and meaningful. Additionally, we will assess the synthesis findings using the CERQual approach [43] to transparently determine how much confidence can be placed in them.

### Phase seven: expressing the synthesis

Findings from the meta-ethnography will be published in a peer-reviewed journal; presented at suitable fora (including surgical and infection conferences and relevant teaching) and made available to patients and members of the public. We will work with key stakeholders and patient representatives to ensure that the media are acceptable to their target audience and disseminated via the most effective channels.

## Discussion

As far as we know, there is no qualitative synthesis that explains how and why contextual factors interact to influence surgical APB. This knowledge is key to the development and implementation of effective, sustainable, interventions to improve practice. The meta-ethnography will develop new theory to broaden understanding of how and why contextual factors act and interact to influence APB amongst surgical teams. Insights will highlight research gaps and inform the development of context-fit quality-improvement interventions to change practice and to improve patient outcomes.

There are several strengths to the research: (1) the protocol has been written with reference to established guidance maximising rigour and transparency; (2) the multi-disciplinary research team brings varied interpretative repertoires and deep experiential knowledge of a wide range of qualitative methods, including qualitative synthesis, which is key in developing theory and explanation [47, 55] and (3) key stakeholders, including patient representatives, will continue to be included throughout the research process to ensure that findings are relevant to healthcare workers and patients who undergo surgery. Furthermore, the involvement of this broad group of stakeholders will support us in disseminating findings via suitable channels to facilitate improvement in practice, for example, one of our stakeholder group is a consultant surgeon and clinical lead for the South West Patient Safety Collaborative.

### Supplementary information

**Additional file 1.** PRISMA-P 2015 Checklist

## Data Availability

Not applicable.

## Appendix

**MEDLINE search strategy**

**Table:**

No.	Search
1	((surgery or surgical or theatre or operat*) adj3 (nurse* or nursing* or doctor* or pharmacist* or trainee* or registrar* or junior* or medic* or physician* or consultant* or team* or group* or profession*)).tw.
2	(surgeon* or anaesthetist* or anesthetist*).tw.
3	exp Surgeons/
4	exp Anesthetists/
5	Perioperative Nursing/
6	Pharmacists/
7	or/1-6
8	(prescrib* or prescrip* or “decision making” or administer or administration).tw.
9	((drug* or medicine* or medication*) adj2 (administ* or utili?ation or error* or use*)).tw.
10	exp Prescriptions/
11	exp Drug Utilization/
12	Inappropriate Prescribing/
13	Prescription Drugs/
14	Drug Prescriptions/
15	Medication Errors/
16	exp Decision Making/
17	or/8-16
18	(antimicrobial* or “anti microbial*” or antibiotic* or “anti biotic*” or antibacterial* or “anti bacterial*” or antiviral* or “anti viral*” or antifungal* or “anti fungal*” or “antiinfective*” or “anti infective*” or antiparasitic* or “anti parasitic*”).tw.
19	Anti-Infective Agents/
20	exp Anti-Bacterial Agents/
21	exp Antifungal Agents/
22	exp Anti-Infective Agents, Urinary/
23	exp Antiparasitic Agents/
24	exp Antiviral Agents/
25	Antibiotic Prophylaxis/
26	Post-Exposure Prophylaxis/
27	Pre-Exposure Prophylaxis/
28	or/18-27
29	7 and 17 and 28
30	interview:.mp.
31	experience:.mp.
32	qualitative:.tw.
33	30 or 31 or 32
34	29 and 33

## Abbreviations

APB: Antimicrobial prescribing behaviour
AMR: Antimicrobial resistance
CASP: Critical Appraisal Skills Programme
NIHR: National Institute for Health Research
SAP: Surgical antimicrobial prophylaxis
